# Elevated plasma glucose links intrafollicular bile acids to altered embryological outcomes in IVF patients

**DOI:** 10.1186/s13048-025-01939-1

**Published:** 2025-12-28

**Authors:** Natascha Berger, Giovanny Rodriguez-Blanco, Katharina Brugger, Bettina Amtmann, Neli Hofer, Martina Kollmann, Irmgard Oreskovic, Katharina Eberhard, Samuele Suraci, Slave Trajanoski, Markus Herrmann, Ursula Hiden, Herbert Fluhr

**Affiliations:** 1 Department of Obstetrics and Gynecology, Medical University of Graz, Auenbruggerplatz 14, Graz, 8036 Austria; 2 Clinical Institute for Medical and Chemical Laboratory Diagnosis CIMCL, Medical University of Graz, Graz, Austria; 3 Research Unit Early Life Determinants (ELiD), Medical University of Graz, Graz, Austria; 4 Core Facility Computational Bioanalytics, Medical University of Graz, Graz, Austria

**Keywords:** Bile acids, Glucose metabolism, In vitro fertilization, Follicular fluid, Embryo development

## Abstract

**Background:**

Human reproduction is intricately linked to systemic metabolic regulation, with disturbances in glucose metabolism adversely affecting fertility outcomes. Beyond their classical role in lipid digestion, bile acids (BAs), cholesterol-derived catabolites, have emerged as bioactive signaling molecules influencing glucose homeostasis and reproductive physiology. Notably, enhanced glycemic control observed after bariatric surgery, BA administration, or BA sequestrant therapy underscores the potential of BAs as regulators of glucose metabolism and as promising biomarkers or therapeutic targets in metabolic disorders. However, most existing studies investigating the endocrine function of BAs within the follicular environment have been limited to animal models, and the relationship between female metabolic status and the intrafollicular BA milieu in humans remains poorly understood. This study investigates whether systemic metabolic states modulate intrafollicular BA composition and examines how these changes may impact reproductive potential and in vitro fertilization outcomes.

**Results:**

Comparative analysis of matched serum and follicular fluid (FF) samples revealed that the primary glycine-conjugated BA, glycochenodeoxycholic acid, was significantly elevated in FF, while the secondary BAs, deoxycholic acid and ursodeoxycholic acid, exhibited marked decreases in FF compared to serum (*p* < 0.001). K-means clustering of intrafollicular BA concentrations identified two distinct groups characterized by significantly different BA levels in both FF and serum (*p* ≤ 0.0001 and *p* < 0.05, respectively). Among the glucometabolic parameters assessed, glucose emerged as a critical determinant of cluster separation, exhibiting the strongest positive correlation with PC1 (*r* = 0.43, *p* < 0.01). A linear regression model further substantiated that intrafollicular BA concentrations were dependent on fasting plasma glucose levels (*p* ≤ 0.01). Elevated plasma glucose also correlated significantly with markers of insulin resistance and metabolic syndrome risk, including HOMA-IR (*r* = 0.39, *p* = 0.01), triglyceride/HDL cholesterol ratio (*r* = 0.33, *p* = 0.04) and leptin/adiponectin ratio (*r* = 0.37, *p* = 0.02). Interestingly, women with higher intrafollicular BA levels showed fewer follicles, zygotes, and blastocysts but experienced higher clinical pregnancy rates following frozen embryo transfer (*p* < 0.05).

**Conclusions:**

These findings reveal a novel link between systemic glucose metabolism and intrafollicular BA homeostasis, suggesting that subtle metabolic imbalances may impair oocyte maturation and embryo development, while potentially enhancing endometrial receptivity or embryo competence in frozen cycles.

**Supplementary Information:**

The online version contains supplementary material available at 10.1186/s13048-025-01939-1.

## Introduction

Infertility affects approximately one in six couples worldwide, with an estimated prevalence of 8–12% among women aged 20–44 years [1]. While parental reproductive aging remains a significant factor, common clinical conditions such as the polycystic ovary syndrome (PCOS), endometriosis, and tubal disease are leading indications for pursuing in vitro fertilization (IVF) [2]. Despite advancements in diagnostics and treatments, approximately 15% of infertile couples are classified with “unexplained infertility” [3]. Emerging evidence underscores the critical role of metabolic health in determining reproductive outcomes [4]. Aberrant glucose metabolism, including hyperinsulinemia, profoundly impacts ovarian function by promoting androgen overproduction, disrupting ovulatory cycles, and reducing endometrial receptivity [5–8]. Hyperglycemia further compromises key aspects of reproduction, including oocyte quality, embryonic development, and implantation success, ultimately leading to poor reproductive success [9, 10]. Concerningly, projections from the World Health Organization indicate a continued rise in the prevalence of obesity and associated metabolic disorders, including type 2 diabetes mellitus (T2DM) [11]. This trend highlights the urgent need for integrated strategies addressing metabolic health to improve reproductive outcomes.

Bile acids (BAs), the end products of cholesterol catabolism, were conventionally recognized for their critical role in facilitating the absorption, transport, and metabolism of dietary lipids and fat-soluble vitamins. Yet, accumulating evidence underscores their function as signaling molecules with a critical role in regulating metabolic homeostasis [12–14]. Particularly, metabolic benefits including augmented insulin sensitivity and more effective postprandial glycemic control observed post-bariatric surgery often coincide with significant elevations in both intestinal and circulating BAs [15, 16]. Conversely, elevated fasting total BA (TBA) levels and alterations in BA pool composition have been observed in obese humans and rodents, with established associations to insulin resistance [17–21]. These conflicting findings highlight the complex and paradoxical relationship between BAs and metabolic changes, suggesting that systemic BA levels represent only a small, transient fraction of the total BA pool. Therefore, investigating BA concentrations in target tissues, such as the ovarian follicle, is crucial to understand their direct effects on the intrafollicular environment. The endocrine function of BAs within the follicle, as well as their potential role in follicular maturation, has been primarily studied in animal models [22, 23]. Whether specific follicular BA profiles are associated with female infertility and how these profiles may be influenced by metabolic alterations remain largely unexplored. In this study, we examined the effects of glucometabolic parameters on circulating and follicular fluid (FF) BA profiles, with the objective to explore if alterations in BA profiles negatively impact IVF outcomes.

## Materials and methods

### Study design and subject characteristics

This pilot study was designed as a prospective, cross-sectional and observational investigation (ClinicalTrials.gov Identifier: NCT06826820). The study adhered to the ethical principles outlined in the Declaration of Helsinki and received approval from the Ethics Committee of the Medical University of Graz (Approval No. 34–459 ex 21/22). Samples were collected at the Division of Gynecological Endocrinology and Reproductive Medicine at the Medical University of Graz between March 2023 and April 2024 during routine visits in the outpatient clinics. Women presented at the fertility center, where a detailed medical history was obtained. During the initial consultation, the treating physician informed and counseled patients about the study, and eligible patients were recruited. A total of 169 patients were screened for study eligibility. Participants eligible for inclusion in the study were women aged 18 to 45 years at the time of sample collection who provided written informed consent to participate. Exclusion criteria specifically targeted individuals with medical conditions deemed by the investigator or treating physician to potentially compromise study integrity or influence outcomes. The indications for an IVF attempt included anatomical factors, endometriosis, PCOS, male factor, unexplained infertility, and other factors such as a history of cancer or uterine abnormalities. All participants underwent a diagnostic cycle followed by ovarian stimulation according to the clinical protocol. Biological samples were collected on the day of oocyte retrieval. For paired analysis (FF with matching serum and plasma samples) 79 women were enrolled in the study. A subset of 40 participants was selected for secondary analysis. Study data were collected and managed using REDCap electronic data capture tools hosted at the Medical University of Graz [24, 25]. Individual body composition, including fat and fat-free mass, was assessed using air displacement plethysmography (BOD POD^®^). Table 1 summarizes the demographic and clinical characteristics of the study cohort, as well as a subgroup (*n* = 40) with documented glucose measurements.

**Table 1:** Baseline characteristics of the study cohort (*n* = 79) and respective subpopulation (*n* = 40)

		Study cohort (*n* = 79)	Subpopulation (*n* = 40)
Subject characteristics	Age (y)	35.0 (32.3–39.1)	34.7 (31.8–38.4)
	BMI (kg/m2)	23.4 (20.8–27.9)	23.9 (21.1–29.1)
	FMI (kg/m2)	7.6 (5.2–12.2)	7.4 (5.4–12.5)
Subfertility	Primary	47 (59)	25 (63)
	Secondary	25 (32)	9 (23)
	Unkown	7 (9)	6 (15)
Diagnosis	Tubal factor	9 (11)	3 (7.5)
	Endometriosis	25 (32)	12 (30)
	PCOS	5 (6)	2 (5)
	Male factor	19 (24)	8 (20)
	Unexplained	17 (22)	9 (22.5)
	Other (cancer, uterine factor)	4 (5)	6 (15)

Demographic and body composition values are reported as median and interquartile range (IQR)

Subfertility diagnosis are presented as N (%)

Abbreviations: *BMI* Body mass index, *FMI* Fat mass index

### Ovarian stimulation and sample collection

Patients were treated with individualized ovarian stimulation per the fertility clinic’s normal practice at the physician´s discretion. In brief, 20% of patients underwent a GnRH agonist protocol and 77% GnRH antagonist protocol. The initial dose of gonadotropin was determined based on the patient’s age, BMI, and ovarian reserve markers. Administered gonadotropins included either recombinant follicle-stimulating hormones (Ovaleap, Theramex; Gonal F, Merck Serono; Rekovelle, Ferring Pharmaceuticals) or highly purified human menopausal gonadotropin (Menopur, Ferring Pharmaceuticals; Meriofert IBSA Institut Biochimique SA). Final oocyte maturation was induced by either an injection of 10,000 IU urinary hCG/250 µg recombinant hCG, or two injections of 0.1 mg triptorelin, as soon as leading follicles of at least 17–20 mm in size were observed on ultrasound scan. Follicular aspiration was performed 36 h post-trigger under transvaginal ultrasound guidance. FF was collected from the first aspirated follicle of each donor, defined as a visually prominent follicle measuring ≥ 18 mm in mean diameter on transvaginal ultrasound and positioned along an accessible retrieval path (mean follicle size 21 ± 3 mm; *n* = 79). FF samples were included only if they contained an oocyte and showed no visible signs of blood contamination. To assess potential blood contamination of FF, hemoglobin concentrations were measured in a randomly selected subset of samples. FF from the leading follicle was analyzed in 29 of the 79 specimens (37%). For each patient, the corresponding peripheral blood hemoglobin concentration was used to estimate the proportion of hemoglobin attributable to blood admixture. Based on these calculations, the mean estimated blood contamination was 0.0621 g/dL ± 0.0847, corresponding to an average contamination rate of 0.47%. Following collection, FF was centrifuged at 810 × g for 10 min at 4 °C to remove cellular debris and contaminants. The resulting supernatant was aliquoted and stored at − 80 °C until further analysis. Luteal phase support included micronized vaginal progesterone alone or in the combination with progesterone given subcutaneously at the physician´s discretion (Cyclogest, Gedeon Richter Plc.; Progedex Farmaceutici Italia Srl). Luteal phase support for fresh embryo transfers started on the evening after oocyte retrieval and continued until a negative pregnancy test or up to 12 weeks of gestation. All frozen embryo transfers cycles used luteal phase support and have been scheduled according to the proposed optimal endometrium preparation as described elsewhere [26].

### IVF laboratory process

The IVF laboratory processes followed the published ESHRE Guidelines (ESHRE - European Society of Human Reproduction and Embryology), with minor modifications [27]. Oocytes were collected and carefully isolated from aspirated FF and placed into GMOPS medium to remove blood and cellular components. The oocyte-cumulus complexes were then transferred into fertilization medium G-IVF. On the same day, a sperm sample was prepared by density gradient and the most motile sperm cells were used for fertilization. On the next day (Day 1) the fertilized oocytes were washed in G1 medium and placed into a freshly prepared culture dish containing 30 µl droplets of G1 culture medium. These droplets were covered with Ovoil to prevent the development of reactive oxygen species, temperature loss, and to maintain a stable pH. On Day 3, the embryos were transferred into a fresh culture dish with 30 µl droplets of G2, again covered with Ovoil. The embryos remained in this medium until fresh embryo transfer (FET) on Day 5 (or Day 6) or until cryopreservation. The embryologist continuously monitored and scored embryo development and quality during culture. The blastocyst transfer was based on Gardner criteria such as expansion (1-6), inner cell mass (A-D) and trophectoderm cells (A-D) and the cleavage stage transfer was based on cell number, symmetry and fragmentation [28, 29]. All reagents and media were purchased from Vitrolife, Göteborg, Sweden. For the cryopreservation of additional blastocysts the VitriFreeze kit (FertiPro, Beernem, Belgium) was used according to the manufacturer’s protocols. In brief, the blastocyst was placed in 300 µl of preincubation solution (PI) for 2 min, in 250 µl of first freezing solution (F1) for 3 min, and finally in a 50 µl droplet of second freezing solution (F2) for 30–60 s. During the last 60 s in F2, the blastocyst was loaded onto the vitrification device (Rapid-i Kit, Vitrolife, Göteborg, Sweden), inserted into the outer straw, and plunged into liquid nitrogen. Vitrified embryos were stored in nitrogen tanks at − 196 °C until further use.

### Blood collection, biochemical analysis, and IVF parameter assessment

Venous fasting blood samples were collected prior to follicular puncture using VACUETTE^®^ tubes (Greiner Bio-One GmbH, Kremsmünster, Austria). Serum and plasma fractions were separated by centrifugation, and glucometabolic markers (glucose, insulin, C-peptide), lipid metabolic parameters (cholesterol, HDL-cholesterol, triglycerides), and hormones (leptin, adiponectin, estrogen, testosterone, and AMH) were analyzed at the Department of Obstetrics and Gynecology and the ISO 15,189 accredited Clinical Institute for Medical and Chemical Laboratory Diagnosis (CIMCL), Medical University of Graz, following manufacturer guidelines. Glycated hemoglobin (HbA1c) was measured in whole blood at the Clinical Institute for Medical and Chemical Laboratory Diagnosis in accordance with manufacturer protocols. Insulin sensitivity was evaluated by the Homeostatic Model Assessment for Insulin Resistance (HOMA-IR), which was calculated as (Fasting Insulin*Fasting Glucose)/22.5. Additionally, IVF parameters were assessed and recorded at the Division of Gynecological Endocrinology and Reproductive Medicine, including antral follicle count (AFC, < 15 mm), follicle count during oocyte retrieval (Follicle Count); number of metaphase II oocytes (MII Oocytes); the number of oocytes with two pronuclei after fertilization (PN2 Zygotes); the number of all fertilized oocytes, including zygotes with one and more than two pronuclei (Fertilization Yield); the total number of embryos that progressed to the blastocyst stage (Blastocyst Count); the number of blastocysts per 2PN (Blastocyst Formation Rate); All parameters reflect outcomes measured within a single IVF treatment cycle. Moreover, clinical pregnancy rates for both fresh and cryopreserved single embryo transfers and live birth rates within one ovarian stimulation cycle were calculated. The live birth rate accounts for all subsequent fresh and frozen embryo transfers derived from a single ovarian stimulation cycle.

### Bile acid (BA) measurements

This targeted method is validated and routinely applied for BA analysis at the University Hospital LKH Graz, in a laboratory accredited according to ISO 15,189 standards. Authentic commercially available standards (Avanti, Merck KGaA, Darmstadt, Germany) were used for method establishment and each compound was tuned and optimized to achieve optimal transitions on the mass spectrometer. Linearity is tested before each batch using calibrators between 0,05 and 100 nmol/ml. External quality control samples, including (patho)physiological samples, are also included in every single run, and the batch is accepted if the values deviate no more than 10% from the target value. Inter- and intra-assay coefficients of variation, evaluated as part of our internal validation for almost all BAs, were better than 10%. Serum and FF samples were processed and analyzed by LC-MS/MS as previously described [30]. Briefly, samples were spiked in with a mixture of internal standards (d4-DCA, d4-LCA, d4-GLCA, d4-GCDCA, and d4-TDCA, each at a concentration of 0.2 nmol) (Sigma Aldrich, Taufkirchen, Germany) and vigorously mixed for 1 min. Proteins were precipitated by addition of 400 µl acetonitrile. After centrifugation (3200 g, 12 min, RT), supernatant evaporated under a flow of nitrogen and reconstituted before analysis. Extracted BAs were separated using a chromatographic gradient using a Nucleoshell C18 reversed phase column (Macherey-Nagel, Düren, Germany). Separation was performed using aqua dest containing 1.2% v/v formic acid and 0.38% w/v ammonium acetate, and elution was carried out using acetonitrile with 1.3% v/v formic acid and 0.38% ammonium acetate. Analysis was performed on Triple Quadrupole mass spectrometer 4500 (Sciex) with an ESI ion source in negative ionisation mode. The limit of quantitation of the mass spectrometer was 0.001 µmol/l for all BA species. Data acquisition and quantitation processing was performed with Analyst Software (Sciex).

### Statistical analysis

SPSS Statistics-Software V.29 (IBM^®^ SPSS Statistics-Software, NY, USA) and Graph Pad Prism 10.4.1 Software (GraphPad Software Inc., CA, USA) were used for statistical analysis and graph plotting. Data are presented as box plots illustrating the median, minimum, maximum, and interquartile range (first and third quartiles). The normality of all data sets was assessed using both the Shapiro-Wilk and Kolmogorov-Smirnov tests. Based on the distribution characteristics, Pearson or Spearman correlation analyses were performed as appropriate. Given the non-parametric distribution of BA species, data were analyzed using the Mann-Whitney U test and the Wilcoxon signed-rank test, with the Bonferroni-Dunn multiple comparison test. K-means clustering was performed using Metaboanalyst 6.0 software [31]. To facilitate data interpretation, the clustering results were visualized using a score plot, which represents cluster memberships on a Principal Component Analysis (PCA) of log-normalized and Pareto-scaled data. Univariate regression analyses was conducted to evaluate the association between fasting plasma glucose and intrafollicular TBA levels (log-normalized). Fisher’s exact test and chi-square test were employed to evaluate IVF outcome parameters, including the proportion of fresh to frozen embryo transfers, clinical pregnancy rates per fresh and frozen embryo transfer, as well as live birth rates, respectively. Notably, as this study was exploratory in nature, no formal sample size calculation was performed.

## Results

### Follicular fluid exhibits reduced secondary and increased glycine-conjugated bile acid levels compared to serum

**Fig. 1:**
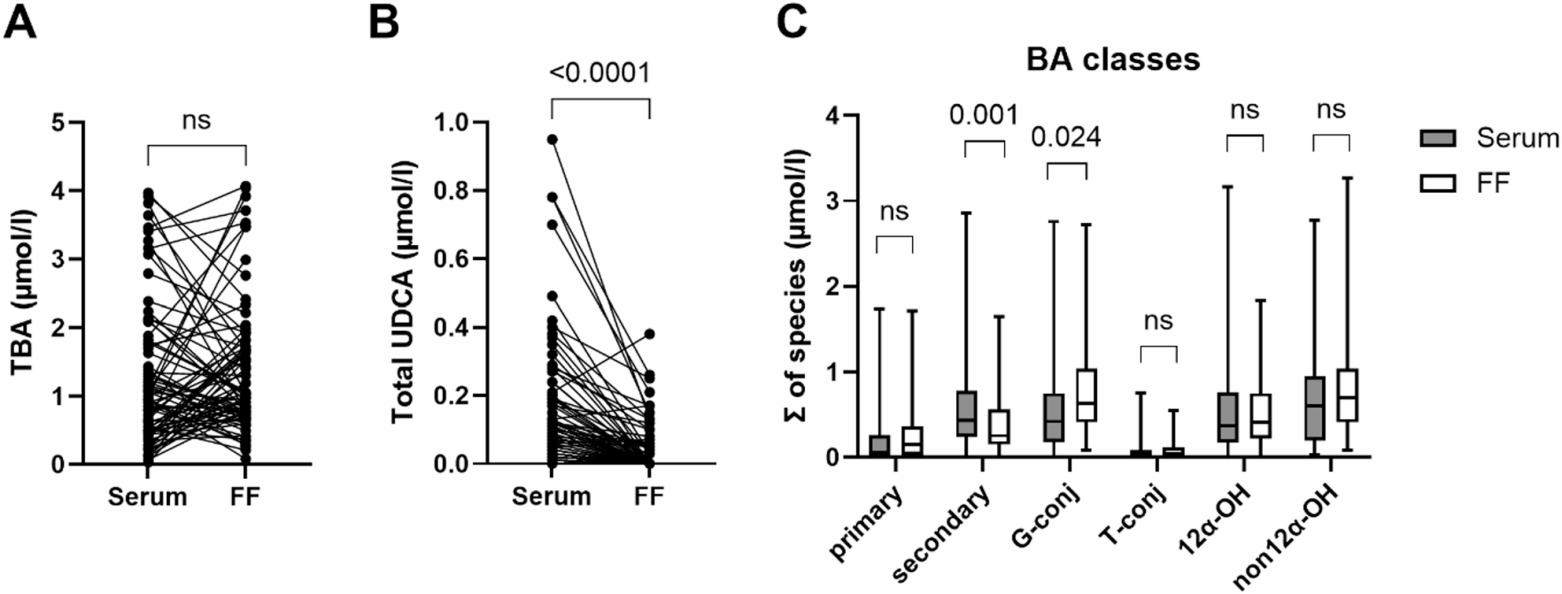
Comparison of bile acid levels in matching human follicular fluid and serum samples **A** Total BA (TBA) and **B** total UDCA levels (µmol/l) were compared between serum and follicular fluid (FF) using the Wilcoxon matched pairs signed rank test **C** Differences between BA classes (µmol/l) in serum and FF were assessed by Wilcoxon signed-rank test and Bonferroni-Dunn post-hoc analysis. The median is represented by a horizontal line. The bottom of the box indicates the 25th percentile, the top the 75th percentile. ns, not significant

We conducted a targeted metabolomic profiling to quantitatively determine a total of 15 BA species in paired serum and FF samples of women undergoing IVF. TBA levels (sum of all measured BA species) did not differ significantly between serum and FF (Fig. 1A). However, the sum of UDCA species (GUDCA, TUDCA, UDCA) was significantly lower in FF compared to serum (*p* < 0.001), primarily due to differences in UDCA levels (*p* < 0.001) (Fig. 1B; Table 2). Analysis of distinct BA classes (sum of BA species assigned to each class) revealed significantly lower concentrations of secondary BAs in FF relative to serum (*p* < 0.001), largely driven by lower levels of DCA and UDCA (*p* < 0.001) (Fig. 1C; Table 2). In contrast, FF contained significantly higher concentrations of glycine-conjugated BAs compared to serum (*p* = 0.02), mainly attributed to elevated levels of GCDCA (*p* < 0.001) (Fig. 1C; Table 2). All BA species and their respective derivatives in serum, with the exception of glycocholic acid (GCA), exhibited direct correlations with their levels in FF (Figure S1). Notably, lithocholic acid (LCA) and its conjugates were undetectable and therefore excluded from further analysis. The complete landscape of BA species in individual paired serum and FF samples is presented in Figure S2.

**Table 2:** Bile acid profiles in matched serum and follicular fluid (FF) samples (*n* = 79)

	Serum	FF	
µmol/l	Median, IQR	Median, IQR	p value
CDCA	0.05 (0.01–0.19)	0.1 (0.03–0.21)	1.00
CA	0 (0–0.06.06)	0.05 (0–0.12.12)	0.79
DCA	0.15 (0.09–0.26)	0.13 (0.05–0.22)	< 0.001
GCA	0.01 (0–0.07.07)	0.03 (0–0.11.11)	1.00
GCDCA	0.28 (0.11–0.46)	0.51 (0.33–0.73)	< 0.001
GDCA	0.09 (0.02–0.21)	0.12 (0.05–0.26)	1.00
GUDCA	0 (0–0.04.04)	0 (0–0.05.05)	1.00
TCA	n.d	n.d.	0.01
TCDCA	0.01 (0–0.06.06)	0.04 (0–0.08.08)	0.29
TDCA	n.d	0 (0–0.03.03)	1.00
TUDCA	n.d	n.d.	1.00
UDCA	0.08 (0.02–0.16)	n.d.	< 0.001

Values are expressed as median and interquartile range (IQR). To assess differences between paired samples multiple testing using Wilcoxon signed-rank test and Bonferroni-Dunn post-hoc was performed; n.d., not detectable; CDCA, chenodeoxycholic acid; CA, cholic acid; DCA, deoxycholic acid; GCA, glycocholic acid; GCDCA, glycochenodeoxycholic acid; GDCA, glycodeoxycholic acid; GUDCA, glycoursodeoxycholic acid; TCA, taurocholic acid; TCDCA, taurochenodeoxycholic acid; TDCA, taurodeoxycholic acid; TUDCA, tauroursodeoxycholic acid; UDCA, ursodeoxycholic acid

### Bile acid clustering in follicular fluid reveals two groups stratified by plasma glucose levels

**Fig. 2:**
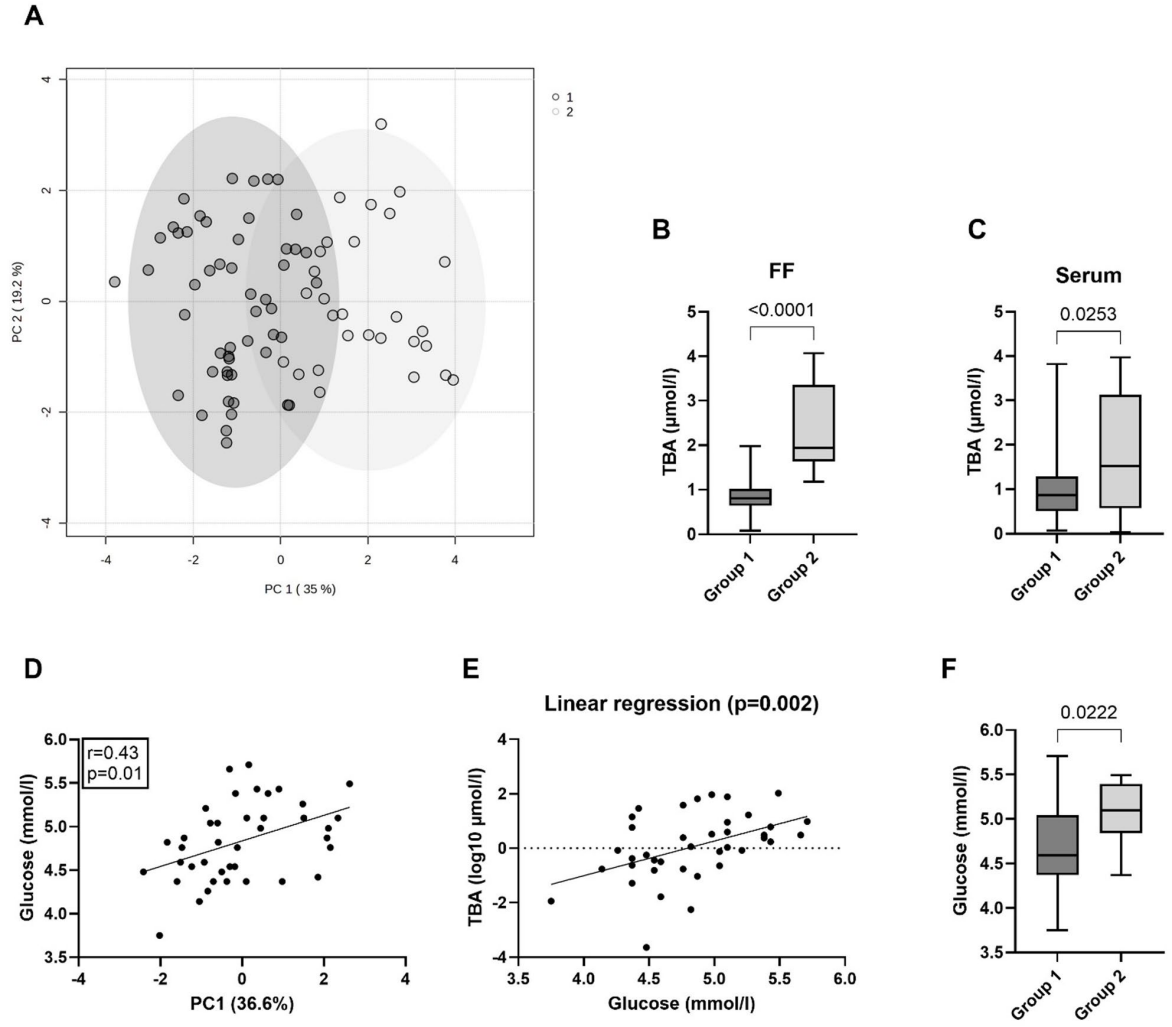
Plasma glucose represents a key driver of intrafollicular BA cluster separation **A** FF BA-dependent group assignment generated by k means clustering is demonstrated on a PCA of log-normalized data (*n* = 79) **B** Total BA (TBA) levels in FF and serum **C** were compared between the two groups using the Mann Whitney U test (Group 1 *n* = 51, Group 2 *n* = 28) **D** Pearson correlation between PC1 and glucose levels (*n* = 40) **E** Univariate regression analysis reveals a significant association between glucose and TBA (log-transformed) (*n* = 40) **F** Glucose levels were compared between groups 1 and 2 using an unpaired t-test (Group 1 *n* = 51, Group 2 *n* = 28). Data are presented as boxplots showing the median, min, max and quartiles

Next, k-means clustering was applied to identify latent groups or patterns in FF based on BA profiles. This analysis revealed two distinct groups, with principal components PC1 and PC2 explaining 35.0% and % of the total variance, respectively (Fig. 2A). Group comparisons demonstrated significant differences in TBA levels between the groups in both FF (*p* < 0.001) and serum (*p* = 0.02) (Fig. 2B, C). The divergence in FF BA profiles was primarily driven by differences in chenodeoxycholic acid (CDCA), its respective glycine and taurine conjugates, and its secondary derivative UDCA along with its glycine conjugates (Table S1). Additionally, glycine conjugates of cholic acid (CA) and DCA significantly contributed to the observed clustering (Table S1). To assess whether metabolic traits influence cluster separation, PC1 and PC2 were correlated with glucometabolic parameters (glucose, HbA1c, insulin, C-peptide, HOMA-IR). Plasma glucose exhibited the strongest correlation with PC1 (*r* = 0.43, *p* = 0.01) (Fig. 2D). A univariate regression analysis demonstrated that fasting plasma glucose was a significant predictor of intrafollicular TBA levels (*p* = 0.002; Fig. 2E, Table S2), with the final model yielding R² = 0.217, and adjusted R² = 0.196, indicating that fasting glucose accounted for approximately 20% of the variance in TBA. Group-wise comparison revealed significantly elevated plasma glucose levels in group 2 compared to group 1 (*p* = 0.02) (Fig. 2F), suggesting a potential link between glucose metabolism and FF BA composition. However, a post-hoc two-tailed t-test (α = 0.05) yielded an effect size of d = 0.84 and a power of 0.70, indicating limited statistical power (data not shown).

To assess potential confounders of the clustering analysis, we evaluated age, BMI, fat mass index (FMI), infertility type (primary vs. secondary), and infertility etiology. None of these parameters differed significantly between groups (Figure S3; Tables S3, S4). However, comparisons of ovarian stimulation regimens revealed significant differences in antral follicle count (AFC), follicle number at oocyte retrieval, and mature (MII) oocyte yield between Groups 1 and 2 when stratified by GnRH agonist versus antagonist protocols (Table S5). Notably, glucose and TBA levels did not differ between protocols, indicating that the primary study outcomes were not confounded by the choice of stimulation regimen.

We further explored the relationship between fasting plasma glucose levels and systemic glucometabolic, lipid metabolic, and hormonal parameters. Correlation analysis revealed significant positive associations between glucose and C-peptide levels (*p* < 0.01, *r* = 0.45), as well as HOMA-IR values (*p* = 0.01, *r* = 0.39) (Table 3). Additionally, elevated glucose levels were significantly associated with higher TG/HDL ratios (*p* = 0.04, *r* = 0.33) and higher leptin-to-adiponectin ratios (*p* = 0.02, *r* = 0.37), which serve as surrogate markers for insulin resistance and metabolic syndrome risk, respectively (Table 3).

**Table 3:** Correlation table of glucose and representative clinical traits of glucose-, lipid-, and hormone- metabolism. Analysis was performed by pearson and spearman correlations, respectively, depending on the normal distribution of data

	Glucose (mmol/l) Vs.	*r* value	*p* value
Glucometabolism	HbA1c (mmol/mol)	0.20	0.25
	C-peptide (nmol/l)	0.45	< 0.01
	Insulin (pmol/l)	0.33	0.40
	HOMA-IR	0.39	0.01
Lipid-/hormone-metabolism	TG/HDL (mmol/l)	0.33	0.04
	Leptin/Adiponectin (ng/ml)	0.37	0.02
	E2/T (ng/ml)	−0.27	0.09

TG/HDL, triglyceride/HDL-cholesterol ratio; E2/T, estradiol/testosterone ratio

### Group 2 exhibits impaired ovarian response and reduced embryological outcomes but higher clinical pregnancy rates in frozen embryo transfers

**Fig. 3:**
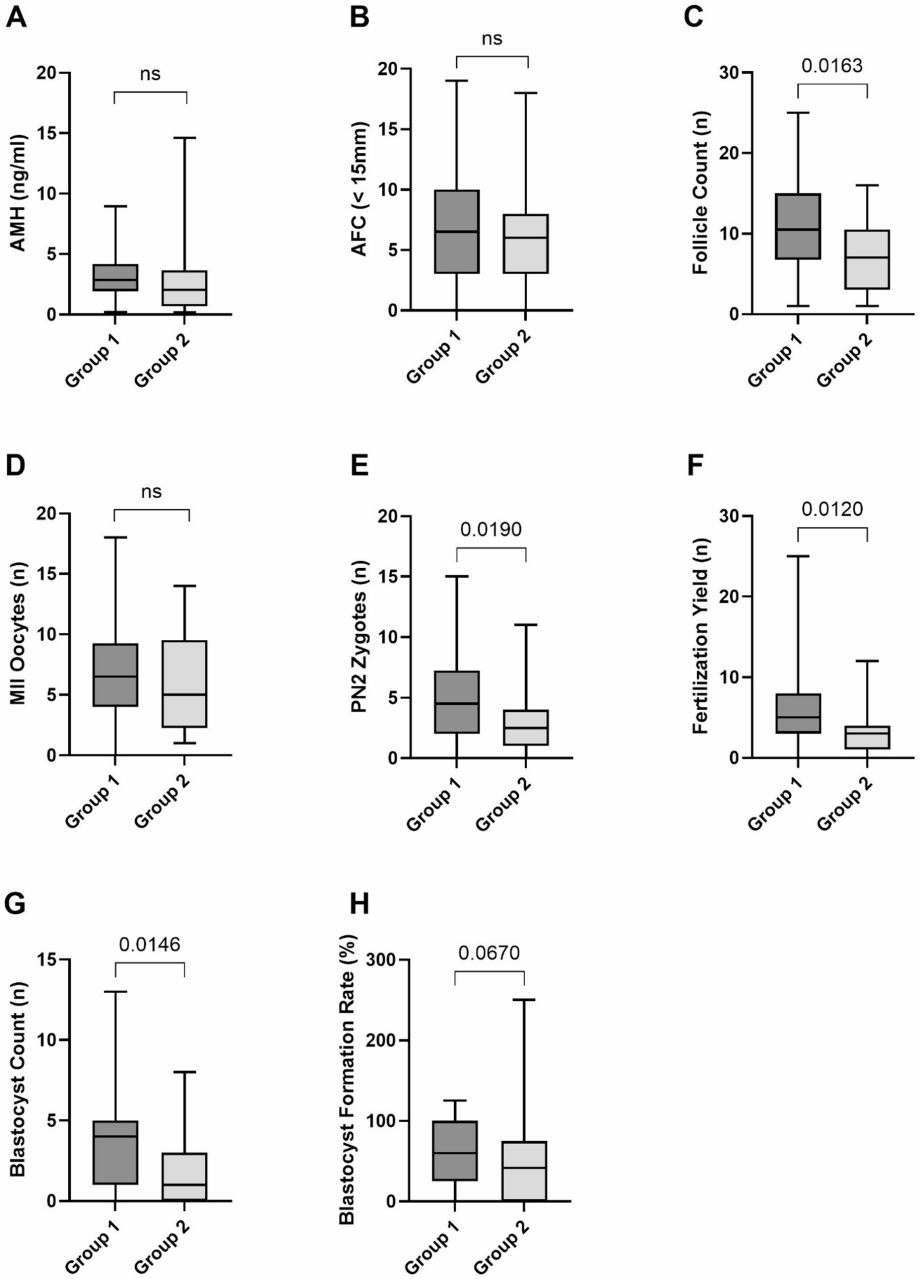
Group 2 exhibits reduced follicle count, zygotes and blastocyst development. Depending on the distribution of the data unpaired t-tests or Mann Whitney U tests were conducted to compare **A** anti-Müllerian hormone (AMH) levels, **B** antral follicle count (AFC), **C** follicle count at oocyte retrieval, **D** the number of metaphase II oocytes (MII), **E **two-pronuclear stage zygotes (PN2), **F** the overall fertilization yield, **G** the number of blastocysts on day five post fertilization and **H **the blastocyst formation rate between the two groups (Group 1 *n* = 51, Group 2 *n* = 28). The median is represented by a horizontal line. The bottom of the box indicates the 25th percentile, the top the 75th percentile. ns, not significant

To evaluate the influence of FF BA concentrations on IVF outcomes, PC1 scores were correlated with key markers of ovarian reserve, oocyte maturity, and embryo development. These markers included AMH, AFC, follicle counts, the number of MII oocytes, PN2 zygotes, the overall fertilization yield, the number of formed blastocysts on day five post fertilization and the blastocyst formation rate. The analysis identified a weak but statistically significant negative correlation between PC1 scores and the number of PN2 zygotes (*p* = 0.02, *r* = −0.26), fertillization yield (*p* = 0.01, *r* = −0.28), blastocyst counts (*p* = 0.01, *r* = −0.28) and the blastocyst formation rate (*p* = 0.04, *r* = −0.23) (Table 4). We further compared these parameters between the two groups characterized by high versus low FF BA levels. Group 2, with elevated FF BA concentrations, exhibited significantly lower numbers of follicles at oocyte retrival (*p* = 0.02), PN2 zygotes (*p* = 0.02), an overall reduced fertilization yield (*p* = 0.01) and blastocysts (*p* = 0.01) compared to Group 1 (Fig. 3). Comparison of IVF outcomes between Group 1 and Group 2 revealed no significant difference in the proportion of fresh versus frozen embryo transfers (0.5 vs. 1.0, *p* = 0.15), clinical pregnancy rates per fresh embryo transfer (FET) (38.1% vs. 38.5%, *p* > 0.9) and live birth rates (28.1% vs. 41.7%, *p* = 0.3) (Table 5). However, clinical pregnancy rates per cryopreserved embryo transfer (CET) were significantly higher in Group 2 compared to Group 1 (72.7% vs. 30.2%, *p* = 0.01) (Table 5). The respective numbers of embryos categorized by quality, along with the corresponding embryo transfer outcomes and live births per group, are presented in Figure S4.

**Table 4:** Correlation table of PC1 and IVF parameters of ovarian reserve, oocyte quality and fertilization. Analysis was performed by pearson and spearman correlations, respectively, depending on the normal distribution of data

	PC1 (36.6%) Vs.	*r* value	*p* value
IVF parameters	AMH (ng/ml)	−0.11	0.37
	AFC (< 15 mm)	−0.01	0.92
	Follicle Count (P)	−0.23	0.06
	MII Oocytes (n)	−0.15	0.18
	PN2 Zygotes (n)	−0.26	0.02
	Fertilization Yield (n)	−0.28	0.01
	Blastocyst Count (n)	−0.28	0.01
	Blastocyst Formation Rate (%)	−0.23	0.04

PC1, principal component 1; AMH, anti-Müllerian hormone; AFC, the total number of antral follicles (< 15 mm in diameter); Follicle Count (P), follicle count follicular puncture during oocyte retrieval; MII Oocytes, number of metaphase II oocytes; PN2 Zygotes, the number of oocytes with two pronuclei after fertilization; Fertilization Yield, number of all fertilized oocytes; Blastocyst Count, the total number of embryos that progressed to the blastocyst stage; Blastocyst Formation Rate, the number of blastocysts per 2PN; All parameters reflect outcomes measured within a single IVF treatment cycle

**Table 5:** Comparison of IVF outcome parameters between group 1 and group 2. No significant differences were observed in the proportion of fresh versus frozen embryo transfers (ET), clinical pregnancy rates per fresh ET (FET), or live birth rates per ET. However, group 2 demonstrated a significantly higher clinical pregnancy rate per cryopreserved ET (CET) (*p* = 0.01). The live birth rate accounts for all subsequent fresh and frozen embryo transfers. Statistical analysis was performed using fisher’s exact test

Group	1	2	*p*- value
Proportion of fresh/frozen ET	0.5	1	0.15
Clinical pregnancies/FET (%)	38.1	38.5	> 0.9
Clinical pregnancies/CET (%)	30.2	72.7	0.01
Live births/ET (%)	28.1	41.7	0.30

## Discussion

This pilot study uncovers a novel relationship between intrafollicular BA profiles, fasting plasma glucose and IVF outcomes. Our findings suggest that elevated fasting plasma glucose levels significantly impact the quantity of intrafollicular BAs. Specifically, we observed a direct association between high fasting plasma glucose and increased intrafollicular BA concentrations. Moreover, women in the high glucose/high BA group demonstrated impaired ovarian and embryological outcomes, as evidenced by reduced follicle counts at oocyte retrieval, lower numbers of PN2 zygotes, and diminished blastocyst formation. These findings suggest a possible metabolic disruption affecting folliculogenesis and oocyte competence, potentially mediated by altered glucose and BA homeostasis within the ovarian microenvironment. Interestingly, despite these early impairments, the same group exhibited higher clinical pregnancy rates following frozen embryo transfers. While previous studies in humans described the presence of BA in FF and investigated associated enzymes, transporters and receptors [32, 33] or focused on reproductive disorders, such as PCOS and diminished ovarian reserve [34, 35], the direct relationship between intrafollicular BA profiles and metabolic derailments has not been described yet.

Upon food intake, BAs are released into the intestine, where they facilitate the intestinal absorption of dietary lipids by emulsifying fats and promoting micelle formation [16]. While the majority of BAs are recycled through the enterohepatic circulation, where they return to the liver for re-secretion, a small fraction escapes into the large intestine or enters the systemic circulation [12]. Emerging evidence highlights that systemic BAs act as steroidal hormones, mediating effects on peripheral tissues beyond the gut and liver, including reproductive tissues. R. A. Nagy and colleagues provided the first detailed characterization of BA species in both serum and FF of IVF patients and further examined the levels of transport molecules and enzymes involved in BA metabolism in human ovarian granulosa cells [32, 33]. Our findings, together with previous studies, point to two primary routes by which BAs appear in FF- passive diffusion from systemic circulation and active transporter-mediated uptake at the ovarian follicle. Notably, they observed that BA concentrations in FF were approximately twice as high as those in serum, suggesting selective accumulation or transport into the follicular environment [32]. In contrast, our findings highlight that primarily glycine conjugated BAs were significantly increased in FF compared to serum. This observation aligns with the predominance of glycine conjugated BAs in humans, which enhances their hydrophilicity, optimizing lipid absorption while rendering these molecules impermeable to cell membranes [36]. The necessity for specific transporters, such as the sodium/BA co-transporter (NCTP) [37], for the uptake of conjugated BAs through the blood-follicle barrier underscores a highly regulated mechanism of BA transport into the follicle. The absence of key BA synthesis enzymes (e.g., CYP7A1) in granulosa cells further indicates that local production within the ovary is unlikely [33]. Instead, expression of known BA importers such as the NTCP, the apical sodium-dependent bile acid transporter (ASBT), and exporters like ABCC3, within granulosa cells, theca cells, and oocytes, support a model of active, regulated transport of BAs from blood into the follicular compartment [33]. Importantly, the expression of BA transporters, such as NTCP, is under regulatory control of the BA nuclear receptor farnesoid X receptor (FXR) [38]. Through modulation of transporter abundance and activity, FXR may critically influence local BA concentrations within the ovarian microenvironment. This, in turn, could alter BA-mediated signaling pathways and thereby impact key processes such as steroid hormone biosynthesis, follicular development, and overall oocyte competence.

During their transit through the gastrointestinal tract, primary BAs undergo microbial metabolism to form secondary BAs. Specifically, CDCA is metabolized into UDCA and LCA, while CA is converted into DCA [36]. Importantly, in healthy physiology, the BA pool typically contains more CDCA than DCA or LCA [36]. Thus, any factor influencing the gut conversion of CA to DCA or CDCA to LCA/UDCA has the potential to enhance pathological processes. The analysis of serum samples in our cohort indicated that CDCA was more abundant than its secondary derivatives, with LCA being undetectable. In contrast, DCA was found to be highly abundant compared to its precursor, CA. Interestingly, we observed a marked decrease in DCA and UDCA levels in FF compared to serum. DCA is typically the predominant colonic BA in humans, but elevated colonic DCA levels are associated with adverse effects, including inflammation and increased epithelial apoptosis [39–41]. Furthermore, a recent study investigated BAs as potential biomarkers in the context of PCOS pathogenesis and reported that serum DCA was linked to testosterone levels and associated with deposition index, fasting, and postprandial insulin levels [34]. These observations suggest a potential protective mechanism limiting its presence in the follicular environment. Conversely, UDCA has been reported to exert cytoprotective and anti-inflammatory effects [42]. Prior studies also associate UDCA derivatives with enhanced cellular division and the development of high-quality embryos on day three post-fertilization [32]. Despite its recognized beneficial roles, we were unable to detect sufficient amounts of UDCA in FF. The fact that hydrophobic BAs, including UDCA, undergo modifications such as glucuronidation, which represents a critical detoxification pathway, underscores the need for expanded profiling of BA species in future studies [43].

To explore latent differences in BA profiles within FF among apparently metabolically healthy IVF patients, k-means clustering was performed, identifying two distinct groups characterized by high versus low BA concentrations. Further analysis of the relationship between BA clustering and glucometabolic markers revealed a significant correlation between fasting plasma glucose levels and PC1, differentiating the clusters. Patients within the high- BA group exhibited significantly elevated fasting plasma glucose levels. Furthermore, elevated plasma glucose was associated with an adverse metabolic profile, including diminished insulin sensitivity, a higher risk factor for metabolic syndrome and an adipokine imbalance- favoring leptin over adiponectin, indicating that individuals in group 2 may have an overall worse metabolic health compared to women in group 1. Several studies demonstrated that elevated circulating BA levels, and specifically certain BA species, including CA, DCA, and CDCA are directly associated with obesity or T2DM [12, 19]. Recent evidence demonstrated that dysregulated FXR and G protein-coupled BA receptor (TGR5) signaling pathways are central to the pathophysiology of T2DM. Overactivation of gut FXR enhances ceramide secretion, suppresses glucagon-like peptide-1 release, and drives gluconeogenesis, while diminished TGR5 signaling exacerbates insulin resistance, inflammation, and systemic metabolic dysfunction [14, 44, 45].

Given the established associations between specific BA species and key reproductive processes such as follicular atresia, embryo development, and live birth rates observed in both human and animal studies [22, 32, 46–48], we sought to explore whether metabolically linked alterations in intrafollicular BA profiles might impact reproductive outcomes. In our analysis of the two identified groups, the high-glucose/high-BA group exhibited a notable reduction in numbers of follicles, fertilized oocytes and impaired blastocyst development. As ligands of nuclear and membrane receptors, BA signaling can influence steroidogenesis, granulosa cell function, and local metabolic pathways that determine oocyte maturation and competence. Granulosa cells play a key role in estradiol synthesis via aromatase, which is essential for follicular growth and differentiation [49]. Studies in murine granulosa cells showed that activating FXR using the synthetic agonist GW4064 reduced aromatase expression [23], while CA treatment in mice reduced ovarian weight, disrupted estrous cycles, and significantly lowered serum progesterone and estradiol, effects mediated by FXR and reversed upon its knockdown [50]. Moreover, as amphipathic molecules, BAs interact with biological membranes, thereby altering membrane packing, cholesterol accessibility, and membrane permeability [13]. Consequently, elevated FF BA concentrations may destabilize early cell division and impair preimplantation embryo morphology. However, the same BA-driven intrafollicular modifications may render surviving embryos more resilient to cryopreservation stress, resulting in higher post-thaw survival and implantation potential. Embryo cryosurvival largely depends on membrane lipid composition, with membranes resistant to phase transitions and ice-induced damage conferring higher post-thaw viability [51]. This dual effect could explain the paradox of impaired early embryo development but improved live birth rates after CET. Furthermore, decoupling of embryo and endometrial synchrony through deferred embryo transfers cycles may allow for improved endometrial receptivity in women who underwent ovarian stimulation protocols [52, 53]. Our observed association between BA and glucose concentrations further suggests a systemic link, since precise glucose metabolism is essential for uterine receptivity, embryo attachment, and decidualization [54]. This effect may be particularly relevant in metabolically altered states, where the hormonal and inflammatory milieu during fresh transfers could impair endometrial function. However, in cases of mild or subclinical metabolic imbalance, such as modest elevations in glucose or insulin, adaptive compensatory mechanisms may be activated to preserve or even enhance endometrial receptivity. These findings suggest that moderate metabolic modulation could play a dual role, limiting oocyte yield while optimizing conditions for implantation, highlighting the nuanced relationship between metabolism and reproductive success.

### Limitations of the study

This study is descriptive in nature and the glucometabolic sub-analysis is constrained by a small sample size, which limits statistical power and increases the risk of Type II error. Therefore, nonsignificant findings should not be interpreted as evidence of no association but may instead reflect insufficient sensitivity to detect true effects. These preliminary observations highlight the need for larger, adequately powered prospective studies to confirm the potential influence of glucose levels on intrafollicular BA composition and to clarify their relevance for IVF outcomes. Furthermore, this analysis was restricted to a single time point, focusing on metabolic parameters and FF collected during oocyte retrieval. Moreover, all participants underwent ovarian stimulation protocols, making it challenging to extrapolate these findings to natural cycles. Exogenous gonadotropins, particularly when administered at supraphysiological doses, induce elevated estradiol concentrations that may alter BA metabolism. Experimental and clinical evidence indicates that such hormonal elevations can modulate hepatic BA synthesis and impair BA transport [55]. These changes can influence circulating BA profiles and may, in turn, impact the follicular microenvironment, potentially affecting oocyte competence and embryo development. While our study was not designed to capture longitudinal changes across the menstrual cycle, we acknowledge that future research should incorporate serial sampling at defined cycle stages and throughout IVF treatment. Furthermore, small subgroup sizes, particularly in the GnRH agonist group, necessitate caution to avoid overinterpretation of protocol-stratified results. We further suggest that future studies should investigate BA dynamics in unstimulated cycles to better delineate the physiological baseline and isolate stimulation-related effects. This would allow a more comprehensive understanding of how dynamic endocrine–metabolic interactions influence BA profiles and their potential role in reproductive outcomes.

## Supplementary Information

Supplementary Material 1. Figure S1. Correlation matrix of BA species and the sum of UDC species (Total UDCA) between serum and FF. Figure S2. Individual BA profiles in matched serum and FF samples. Figure S3. Evaluation of potential confounding factors influencing group allocation. Figure S4. Presentation of IVF outcome parameters stratified by group. Table S1. Group 2 exhibits significantly higher intrafollicular BA concentrations compared to group 1. Table S2. Regression coefficients for the dependent variable TBA (log-normalized). Table S3. Distribution of clinical diagnosis per group. Table S4. Association between infertility diagnosis and cluster generation, evaluated using Pearson’s chi-square test. Table S5. Comparison of metabolic and IVF parameters between GnRH agonist and antagonist protocols within Groups 1 and 2. Differences were evaluated using Student’s t-test.

## Data Availability

All unprocessed metabolomics data has been deposited at Zenodo at [https://doi.org/10.5281/zenodo.15386725] and is publicly available. Any additional information required to reanalyze the data reported in this paper is available from the lead contact upon request.
