# Supplementary material for: Elevated plasma glucose links intrafollicular bile acids to altered embryological outcomes in IVF patients
Source: J Ovarian Res. 2025 Dec 28;19:39. doi: 10.1186/s13048-025-01939-1 (PMC12866166; doi:10.1186/s13048-025-01939-1)
Supplement: Supplementary file 1 — Supplementary Material 1. Figure S1. Correlation matrix of BA species and the sum of UDC species (Total UDCA) between serum and FF. Figure S2. Individual BA profiles in matched serum and FF samples. Figure S3. Evaluation of potential confounding factors influencing group allocation. Figure S4. Presentation of IVF outcome parameters stratified by group. Table S1. Group 2 exhibits significantly higher intrafollicular BA concentrations compared to group 1. Table S2. Regression coefficients for the dependent variable TBA (log-normalized). Table S3. Distribution of clinical diagnosis per group. Table S4. Association between infertility diagnosis and cluster generation, evaluated using Pearson’s chi-square test. Table S5. Comparison of metabolic and IVF parameters between GnRH agonist and antagonist protocols within Groups 1 and 2. Differences were evaluated using Student’s t-test. [file 13048_2025_1939_MOESM1_ESM.docx]

**Supplemental information**


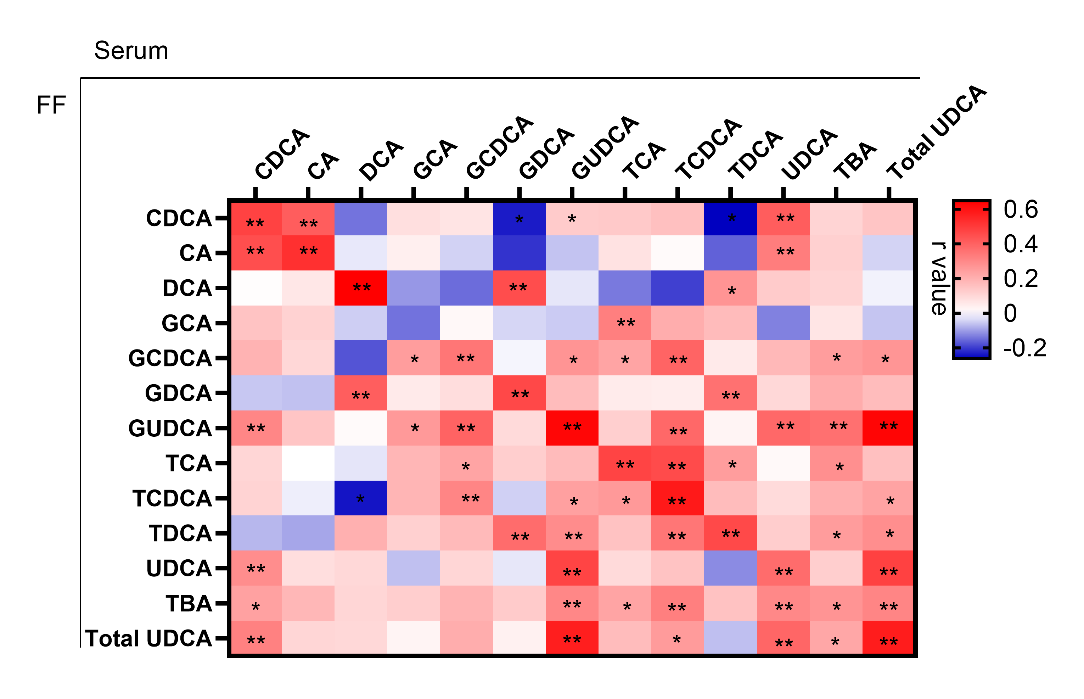


Figure S1: Correlation matrix of BA species and the sum of UDC species (Total UDCA) between serum and FF. Spearman′s correlation coefficient (r value) is presented for each pair of parameters. Significant values are presented: * P ≤ 0.05; ** P ≤ 0.01


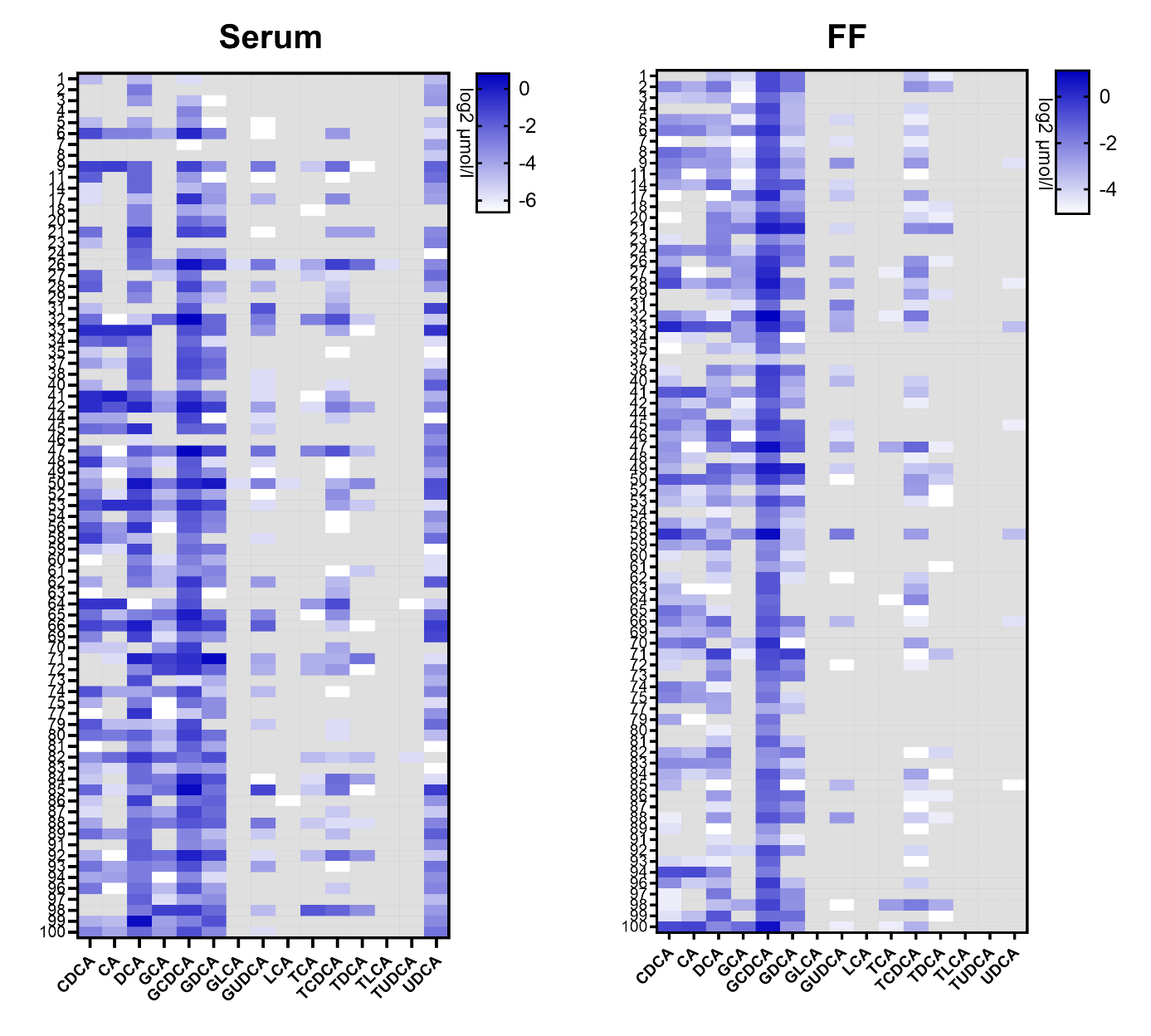


Figure S2: Individual BA profiles in matched serum and FF samples (n=79). BA concentrations were log-transformed for visualization. Grey indicates missing values. CDCA, chenodeoxycholic acid; CA, cholic acid; DCA, deoxycholic acid; GCA, glycocholic acid; GCDCA, glycochenodeoxycholic acid; GDCA, glycodeoxycholic acid; GLCA, glycolithocholic acid; GUDCA, glycoursodeoxycholic acid; LCA, lithocholic acid; TCA, taurocholic acid; TCDCA, taurochenodeoxycholic acid; TDCA, taurodeoxycholic acid; TLCA, taurolithocholic acid; TUDCA, tauroursodeoxycholic acid; UDCA, ursodeoxycholic acid;


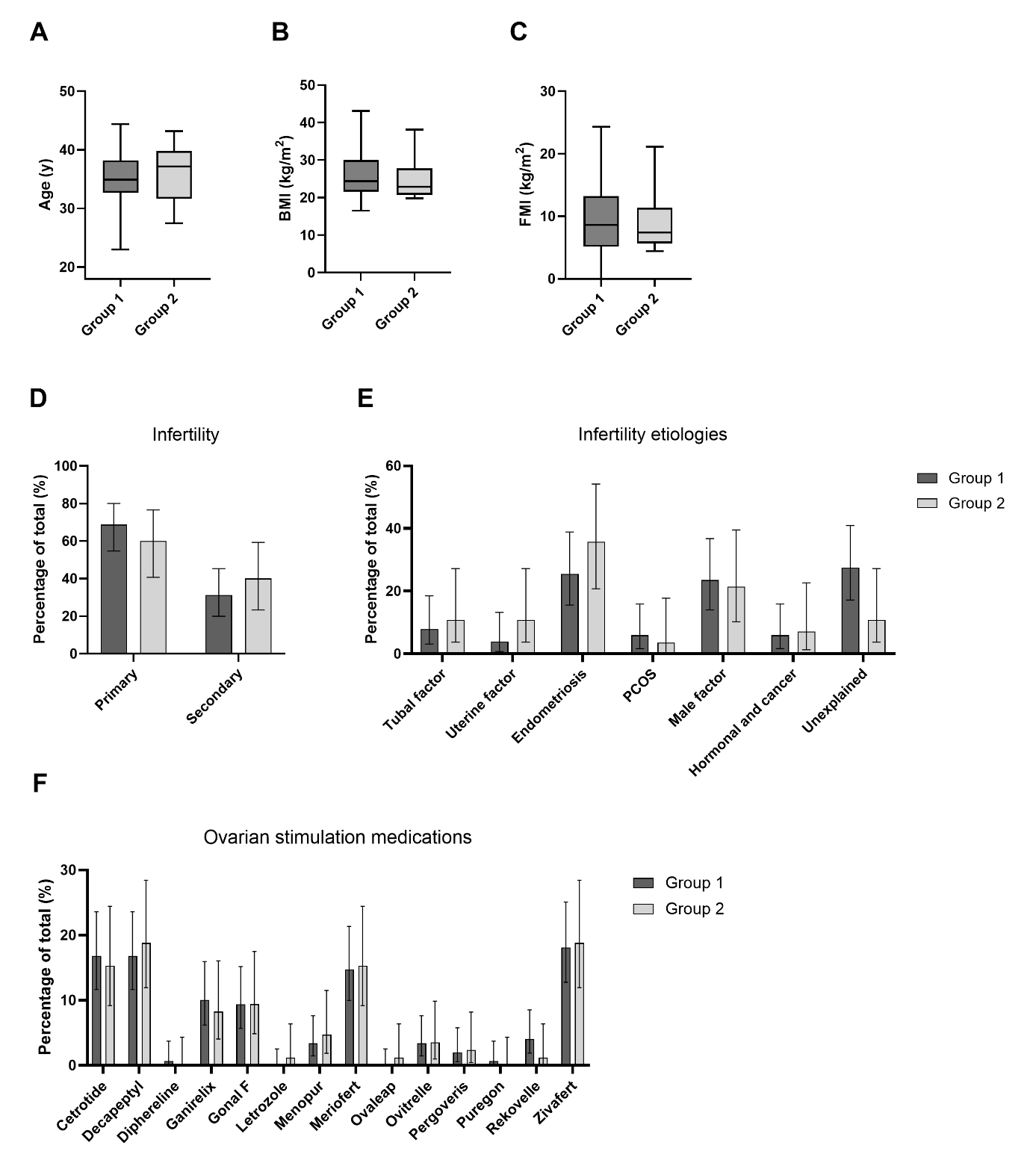


Figure S3: Evaluation of potential confounding factors influencing group allocation. Unpaired t-tests (A–C) and parts-of-whole analyses (D–F) were conducted to assess differences between Group 1 (n=51) and Group 2 (n=28) across relevant demographic and clinical parameters.


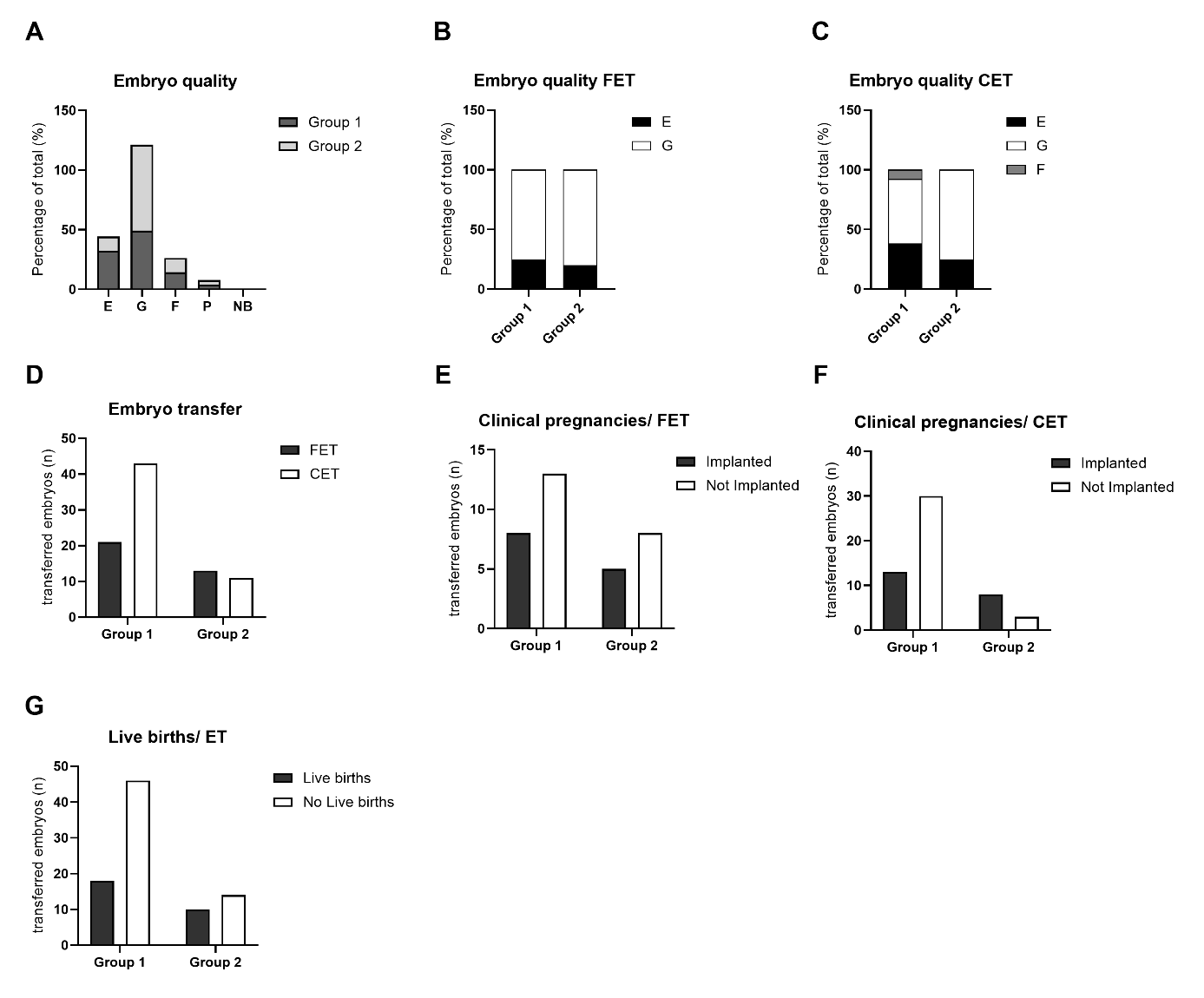


Figure S4: Presentation of IVF outcome parameters stratified by group. (A) Distribution of embryo quality grades per group. Blastocyst quality was assessed by expansion stage, inner cell mass and trophectoderm. (E) Excellent embryos were fully expanded/hatched. (G) Good embryos showed advanced expansion. (F) Fair embryos displayed moderate expansion and (P) poor embryos had low expansion. (NB) Non-blast embryos failed to reach the blastocyst stage by Day 6. In Group 1, 77 embryos were evaluated: 25 (32.5%) were classified as excellent, 38 (49.4%) as good, 11 (14.3%) as fair, and 3 (3.9%) as poor quality. In Group 2, 25 embryos were evaluated: 3 (12%) were excellent, 18 (72%) good, 3 (12%) fair, and 1 (4%) poor. (B, C) Comparison of embryo quality in fresh and cryopreserved embryo transfers (FET and CET). (B) In Group 1, FET embryos included 2 (25%) of excellent and 5 (75%) of good quality; in Group 2, 1 (20%) was excellent and 4 (80%) good. (C) In Group 1, CET embryos included 5 (38.5%) excellent, 7 (53.8%) good, and 1 (7.7%) fair quality, whereas in Group 2, 2 (25%) were excellent and 6 (75%) good. (D) Proportion of fresh and cryopreserved embryo transfers (FET and CET). (E, F) Clinical pregnancy rates following fresh and cryopreserved transfers, respectively. (G) Live birth rates per embryo transfer, including both fresh and frozen transfers within one cycle. Group 1, n=51; Group 2, n=28;

Table S1: Group 2 exhibits significantly higher intrafollicular BA concentrations compared to group 1. A pseudocount of 0.001 (detection limit) was added to all values for fold change calculation to avoid division by zero. To compare BA species and the sum of UDCA species (Total UDCA) between Group 1 and Group 2, Mann Whitney U testing followed by Bonferroni-Dunn method was applied.

|  | Group 1 | Group 2 |  |  |
| --- | --- | --- | --- | --- |
| µmol/l | Median, IQR | Median, IQR | Fold change | p value |
| CDCA | 0.06 (0.00-0.15) | 0.20 (0.08-0.34) | 3.21 | <0.01 |
| CA | 0.00 (0.00-0.08) | 0.12 (0.00-0.23) | 121 | 0.03 |
| DCA | 0.08 (0.03-0.15) | 0.22 (0.13-0.36) | 2.67 | <0.001 |
| GCA | 0.00 (0.00-0.05) | 0.12 (0.04-0.21) | 121 | <0.001 |
| GCDCA | 0.38 (0.25-0.51) | 0.74 (0.59-1.30) | 1.93 | <0.001 |
| GDCA | 0.08 (0.00-0.16) | 0.28 (0.13-0.36) | 3.41 | <0.001 |
| GUDCA | n.d | 0.06 (0.01-0.13) | 61 | <0.001 |
| TCA | n.d | n.d | 1 | 1.00 |
| TCDCA | 0.03 (0.00-0.06) | 0.08 (0.04-0.17) | 2.61 | <0.01 |
| TDCA | n.d | 0.00 (0.00-0.04) | 1 | 0.69 |
| TUDCA | n.d | n.d | 1 | 1.00 |
| UDCA | n.d | 0.00 (0.00-0.09) | 1 | 0.02 |
| Total UDCA | n.d | 0.06 (0.01-0.13) | 61 | <0.001 |

Values are expressed as median and interquartile range (IQR). n.d., not detectable; CDCA, chenodeoxycholic acid; CA, cholic acid; DCA, deoxycholic acid; GCA, glycocholic acid; GCDCA, glycochenodeoxycholic acid; GDCA, glycodeoxycholic acid; GUDCA, glycoursodeoxycholic acid; TCA, taurocholic acid; TCDCA, taurochenodeoxycholic acid; TDCA, taurodeoxycholic acid; TUDCA, tauroursodeoxycholic acid; UDCA, ursodeoxycholic acid;

Table S2: Univariate linear regression of fasting glucose predicting total bile acids (TBA, log-transformed). Model fit indices are reported, including R, R², adjusted R², SE, F-statistics, degrees of freedom (df1, df2) and p-value.

| Model | R | R² | R²_adjusted_ | SE | F | df1 | df2 | p-value |
| --- | --- | --- | --- | --- | --- | --- | --- | --- |
|  | 0.466 | 0.217 | 0.196 | 0.331 | 10.532 | 1 | 38 | 0.002 |

|  | B | SEB | Beta | p-value |
| --- | --- | --- | --- | --- |
| Intercept | -1.845 | 0.575 |  | 0.003 |
| fasting plasma glucose (mmol/l) | 0.385 | 0.119 | 0.466 | 0.002 |

Note: Model with predictors (Constant) and fasting plasma glucose (mmol/l) and with the depend variable TBA; SE =Standard error; B=unstandardized regression coefficient; SEB =Standard error of the coefficient; Beta=standardized coefficient; p-value=significance level

Table S3: Distribution of clinical diagnosis per group.

|  |  |  | Cluster * Diagnosis Cross Table | | | | | | | |
| --- | --- | --- | --- | --- | --- | --- | --- | --- | --- | --- |
|  |  |  | Anatomical factor | Endometriosis | Hormonal and cancer | Male factor | PCOS | Unexplained infertility | Uterine factor | Total |
| Group | 1 | N | 4 | 13 | 3 | 12 | 3 | 14 | 2 | 51 |
|  |  | % of Group | 7.8% | 25.5% | 5.9% | 23.5% | 5.9% | 27.5% | 3.9% | 100.0% |
|  | 2 | N | 3 | 11 | 2 | 6 | 1 | 3 | 2 | 28 |
|  |  | % of Group | 10.7% | 39.3% | 7.1% | 21.4% | 3.6% | 10.7% | 7.1% | 100.0% |
| Total |  | N | 7 | 24 | 5 | 18 | 4 | 17 | 4 | 79 |
|  |  | % of Total | 8.9% | 30.4% | 6.3% | 22.8% | 5.1% | 21.5% | 5.1% | 100.0% |

Table S4: Association between infertility diagnosis and cluster generation, evaluated using Pearson’s chi-square test.

|  | Value | df | p-value |  |  |
| --- | --- | --- | --- | --- | --- |
| Pearson's chi-square | 4.295^a^ | 6 | 0.637 |  |  |
| Likelihood ratio | 4.526 | 6 | 0.606 |  |  |
| Number of valid cases | 79 |  |  |  |  |
| a. 8 cells (57.1%) have an expected frequency of less than 5. The minimum expected frequency is 1.42. | | | | | |

Table S5: Comparison of metabolic and IVF parameters between GnRH agonist and antagonist protocols within Groups 1 and 2. Differences were evaluated using Student’s t-test.

|  | GnRH agonist |  |  | GnRH antagonist |  |  |  |
| --- | --- | --- | --- | --- | --- | --- | --- |
|  | Mean | ± SD | N | Mean | ± SD | N | p value |
| **Glucose (mmol/l)** |  |  |  |  |  |  |  |
| Group 1 | 4.59 | - | 1 | 4.72 | 0.46 | 25 | >0.1 |
| Group 2 | 5.25 | 0.25 | 4 | 5.03 | 0.33 | 9 | >0.1 |
| **TBA (µmol/l)** |  |  |  |  |  |  |  |
| Group 1 | 0.81 | 0.13 | 9 | 0.86 | 0.44 | 41 | >0.1 |
| Group 2 | 2.69 | 1.32 | 6 | 2.20 | 0.85 | 20 | >0.1 |
| **AMH (ng/ml)** |  |  |  |  |  |  |  |
| Group 1 |  |  |  |  |  |  |  |
| Group 2 |  |  |  |  |  |  |  |
| **AFC (<15mm)** |  |  |  |  |  |  |  |
| Group 1 | 2.33 | 1.63 | 6 | 7.93 | 4.32 | 27 | <0.01 |
| Group 2 | 3.80 | 3.27 | 5 | 7.17 | 4.5 | 18 | >0.1 |
| **Follicle count (P)** |  |  |  |  |  |  |  |
| Group 1 | 6.56 | 5.75 | 9 | 11.94 | 5.23 | 33 | <0.05 |
| Group 2 | 3.83 | 2.40 | 6 | 8.89 | 4.40 | 18 | <0.05 |
| **MII Oocytes (n)** |  |  |  |  |  |  |  |
| Group 1 | 4.89 | 5.50 | 9 | 7.78 | 4.12 | 40 | 0.08 |
| Group 2 | 2.17 | 1.60 | 6 | 6.75 | 3.99 | 20 | <0.05 |
| **PN2 Zygotes (n)** |  |  |  |  |  |  |  |
| Group 1 | 1.83 | 1.17 | 6 | 3.90 | 3.34 | 20 | 0.08 |
| Group 2 | 4 | 4.80 | 9 | 5.55 | 3.50 | 40 | >0.1 |
| **Fertilization Yield (n)** |  |  |  |  |  |  |  |
| Group 1 | 1.33 | 1.03 | 6 | 4.40 | 3.56 | 20 | >0.05 |
| Group 2 | 4.11 | 5.08 | 9 | 6.23 | 4.42 | 40 | >0.1 |
| **Blastocyst Count (n)** |  |  |  |  |  |  |  |
| Group 1 | 0.67 | 0.82 | 6 | 2.10 | 2.61 | 20 | >0.1 |
| Group 2 | 2.67 | 3.46 | 9 | 3.80 | 3.07 | 41 | >0.1 |
| **Blastocyst Formation Rate (%)** |  |  |  |  |  |  |  |
| Group 1 | 37.50 | 49.37 | 6 | 44.94 | 58.49 | 20 | >0.1 |
| Group 2 | 44.16 | 45.00 | 9 | 62.16 | 36.34 | 41 | >0.1 |
